# A Review of Past Research and Some Future Perspectives Regarding Titanium Alloys in Biomedical Applications

**DOI:** 10.3390/jfb16040144

**Published:** 2025-04-18

**Authors:** Alex-Barna Kacsó, Ildiko Peter

**Affiliations:** 1 Doctoral School of I.O.S.U.D., George Emil Palade University of Medicine, Pharmacy, Science, and Technology of Targu Mures, Gheorghe Marinescu 38, 540142 Târgu Mures, Romania; alex.kacso@umfst.ro; 2 Department of Industrial Engineering and Management, George Emil Palade University of Medicine, Pharmacy, Science, and Technology of Târgu Mures, Gheorghe Marinescu 38, 540142 Târgu Mures, Romania

**Keywords:** titanium-based alloys, nanoparticles, biomedical application, smart materials

## Abstract

This review paper provides a comprehensive synthesis of the current advancements in investigations of different titanium-based alloys, including pure titanium, commercially available Ti6Al4V, and modified alloys, such as Ti-Nb-Zr-Fe alloys, for biomedical applications. Several researchers have explored the effects of alloying elements and processing techniques on enhancing the mechanical, chemical, and biological properties of these materials. Ti-Nb-Zr-Fe alloys are of particular interest due to their potential to address critical requirements in medical applications, including reduced Young’s modulus, superior corrosion resistance, biocompatibility, and mechanical strength. Despite substantial progress, the detailed mechanisms for optimizing these properties remain underexplored in the current literature. The main objective of the present review paper is to emphasize the importance of ongoing investigations aimed at overcoming challenges such as biocompatibility concerns, fatigue resistance, and wear under biological conditions. By critically analyzing existing data, this study highlights gaps in knowledge and identifies opportunities for advancing research on these alloys. Specifically, this review paper highlights the need for targeted studies to reduce the Young’s modulus and improve other critical characteristics of Ti-Nb-Zr-Fe alloys to better meet the demands of orthopedic implants, dental prosthetics, and cardiovascular devices. Even if the current scientific literature is ample on this topic, we consider that through this review we can positively contribute to the collective effort in this field trying to fill some gaps, including some updates on the topic, time frames, advantages, and limitations, and pave the way for further advancements that could revolutionize biomedical implant technology. The review encompasses studies performed over the last 5 decades, specifically from 1975 to 2025, to ensure the inclusion of the most relevant and up-to-date research. This approach aims to highlight the significant progress made while situating the findings within the broader context of ongoing investigations.

## 1. Introduction

The ongoing demand for advanced materials in biomedical engineering has led to extensive research into titanium-based alloys, particularly Ti-Nb-Zr-Fe (TNZF) alloys, due to their remarkable properties, such as high mechanical strength, excellent corrosion resistance, and outstanding biocompatibility [1]. Biomedical applications demand materials that meet strict requirements, including mechanical strengths ranging from 600 MPa to over 1200 MPa to withstand physiological loads; exceptional corrosion resistance, with rates as low as 0.01 mm/year; and critical biocompatibility to prevent adverse reactions in the body. Moreover, these alloys must exhibit a low elastic modulus, ideally between 40 and110 GPa, to match bone tissue and reduce stress shielding while promoting osteointegration. Furthermore, surface modifications have demonstrated their effectiveness in achieving bone-to-implant contact rates exceeding 80% in clinical studies, enhancing integration with surrounding bone tissue [2].

While titanium (Ti) alloys have demonstrated their reliability in various biomedical applications, their limitations—such as stress shielding due to mismatched elastic modulus and challenges in long-term biocompatibility—have prompted ongoing research efforts aimed at addressing these concerns [3,4,5]; for these reasons, the research in this field is continuously evolving to find new and better solutions. Researchers are increasingly exploring novel solutions, sometimes starting from the existing possibilities and modifying them to optimize Ti alloys for orthopedic, dental, and cardiovascular applications. There are many possibilities to achieve such enhancements, and here, some suggestions will be briefly presented.

Adding alloying elements such as Nb, Zr, and Fe has been shown to significantly improve the biocompatibility and corrosion resistance of Ti alloys, ensuring their reliability for long-term implantation [6].

In addition to alloying elements, nanostructured materials have demonstrated transformative effects on osteointegration, mechanical strength, and wear resistance. For instance, hydroxyapatite (HA) nanoparticles enhance bonding with bone tissue, while surface coatings such as Mg- or Zn-doped HA exhibit increased biomineralization and hydrophobicity, with contact angles ranging from 90° to 110° [7]. Tubular oxide layers and HA coatings on porous Ti alloys like Ti_13_Nb_13_Zr further improve bone-to-implant contact rates by over 80% [8]. Similarly, zirconia (ZrO_2_) nanoparticles enhance wear resistance and stability [9], while silicon carbide (SiC) nanoparticles increase the mechanical strength of alloys, with reported tensile strengths reaching up to 1200 MPa [10]. Beta Ti alloys incorporating elements such as Nb, Zr, Ta, Si, and Fe achieve high tensile strength, excellent corrosion resistance in simulated body fluids, and contact angles of 70–90°, depending on surface treatments [11].

Building on these material improvements, researchers have integrated sensors into Ti-based implants, enabling real-time monitoring of physiological parameters such as temperature, pH levels, and mechanical stress. For instance, low-profile antennas in TiNb-alloy-based devices allow for continuous bio-monitoring and health management [12]. Deep implantable antennas have demonstrated the transmission of biological signals from biosensors, showcasing their practicality for continuous monitoring [13]. This approach has paved the way for smart biomaterials that respond to environmental stimuli, including light, temperature, or pH, and facilitate applications like tissue engineering and diagnostics [14].

To further advance medical technology, recent studies have focused on the surface interactions of Ti-based alloys, such as Ti-29Nb-13Ta-4.6Zr (TNZT) and commercially pure Ti (CP-Ti), with biological fluids under inflammatory conditions. Real-time analysis using techniques like atomic force microscopy and spectroelectrochemistry provides insights into degradation rates and oxidation mechanisms, which are crucial for developing corrosion-resistant materials [15]. Severe plastic deformation techniques, such as high-pressure torsion, have reduced grain sizes to below 100 nm, improving yield strength by 30–50% and enhancing fatigue resistance [16]. Porous Ti alloys, with porosity levels of 30–70%, offer optimal conditions for bone ingrowth and reduced stress shielding, closely matching the elastic modulus of natural bone [17]. Furthermore, the antibacterial properties of Ti nanotubes significantly reduce implant-related infection risks by up to 95%, as demonstrated in in vitro studies [18]. TNZF alloys, particularly those enhanced with advanced techniques and embedded sensors, represent a promising step toward next-generation biomedical implants by addressing both material challenges and smart functionality requirements [19,20,21,22].

The key composition and mechanical properties of Ti alloys discussed throughout this introduction are summarized in Table 1 and Table 2, respectively. These tables provide an overview of the elemental composition and key performance characteristics of TNZF and other Ti alloys, offering a concise reference for the advancements highlighted.

This review synthesizes the current advancements in Ti-based biomaterials with a focus on their composition, properties, and applications. By analyzing the integration of nanostructures and sensors into TNZF alloys, it aims to elucidate the mechanisms behind these enhancements and identify challenges for future research, offering insights into the transformative potential of these materials in improving patient care.

## 2. A Brief History of Ti Alloys in Biomedical Applications

In this chapter, we delve into the evolution of biomaterials, beginning with Ti alloys as early pioneers and tracing the advancements that have led to the discovery of new materials for improved medical applications.

### 2.1. 1950s–1960s: Early Developments

Ti’s medical use began in the 1950s when Per-Ingvar Brånemark demonstrated its ability to bond with bone tissue, introducing it as a material for dental and orthopedic implants [23]. Researchers focused on CP-Ti for its combination of biocompatibility, with a corrosion rate typically less than 0.005 mm/year, and mechanical properties, such as a tensile strength of approximately 240–350 MPa. Its Young’s modulus, ranging between 100 and 105 GPa, further contributed to its suitability for early biomedical applications. Techniques like plasma electrolytic oxidation (PEO), achieving oxide layer thicknesses up to 10 μm, emerged during this time, significantly enhancing CP-Ti’s performance in physiological environments [24]. While CP-Ti proved to be highly effective, research has continued over the years to improve its Young’s modulus, driving further advancements in the development of titanium-based materials for biomedical applications.

### 2.2. 1970s: Introduction of Ti-6Al-4V Alloy

The 1970s marked a significant development in biomedical materials with the introduction of the Ti-6Al-4V alloy. Made of Ti, Al, and V, this alloy offered improved mechanical strength, with a tensile strength of approximately 895–1170 MPa and a yield strength of 828–1100 MPa. Its Young’s modulus ranges between 105 and 120 GPa, providing better load-bearing capabilities compared to CP-Ti. Additionally, its corrosion rate is typically less than 0.01 mm/year, making it highly resistant to degradation in physiological environments. These properties addressed the growing demands of orthopedic applications, particularly for load-bearing implants such as hip and knee replacements.

**Figure 1:**
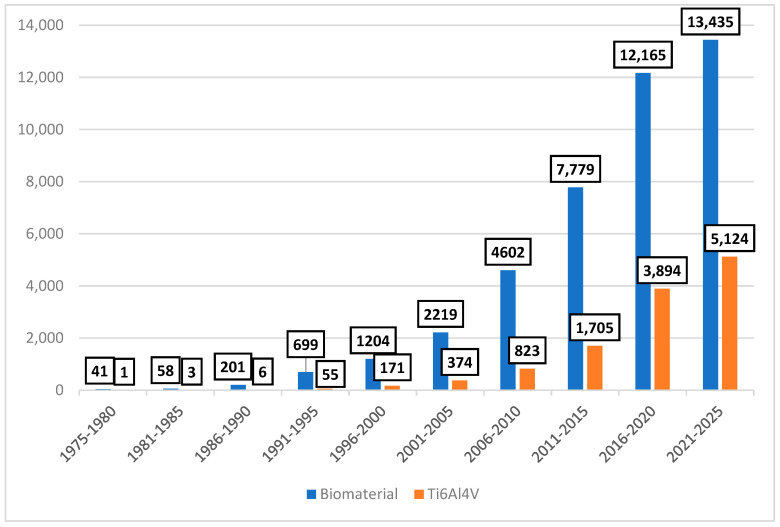
Research trend. The number of papers per year related to biomaterial and Ti6Al4V over the last 50 years. (Data have been collected from Web of Science).

Figure 1 illustrates the steady increase in scientific publications on biomaterials and Ti6Al4V over the last 50 years, highlighting the growing research interest and innovation in biomedical applications together with the necessity for further studies and results, considering the acquired knowledge till now.

The research during this period explored ways to enhance the properties of Ti-6Al-4V. Surface modifications, such as anodized Ti dioxide (TiO_2_) nanotubes with diameters ranging from 30 to 100 nm, were found to improve cell adhesion and viability, making the alloy more compatible with osteoblasts and chondrocytes [25]. Manufacturing advancements, such as selective laser melting (SLM), optimized implant design by tailoring porosity to ranges of 30–70% and mechanical properties with tensile strengths of 850–1100 MPa, further improving implant performance [26]. The use of laser-sintered and CNC-milled Ti-6Al-4V components for dental and prosthetic restorations aligned with emerging concepts like Dentistry 4.0, offering precise customization and enhanced biocompatibility, with dimensional tolerances as low as 10 μm [27].

Clinical studies demonstrated Ti-6Al-4V’s ability to meet the mechanical demands of joint replacements, establishing it as a reliable material in orthopedic implants. Innovations such as incorporating nano-hydroxyapatite (n-HAp) through electrical discharge machining (EDM) improved bioactivity and implant integration, further advancing its functionality [28].

### 2.3. 1980s–1990s: Expanding Applications and Research

The 1980s and 1990s saw significant advancements in the use of Ti alloys for biomedical applications, addressing specific clinical challenges, and researchers developed new alloy compositions. For instance, Ti-6Al-7Nb was introduced as an alternative to Ti-6Al-4V to mitigate concerns about the cytotoxicity of V. Studies showed that Ti-6Al-7Nb not only retained the mechanical strength and corrosion resistance of Ti-6Al-4V but also exhibited lower thrombogenicity and reduced bacterial biofilm formation, improving its biocompatibility [29].

During this period, innovations in surface modification techniques played a vital role in enhancing Ti implants. Methods such as plasma spraying and anodization improved osseointegration and wear resistance. The application of HA coatings became widespread, facilitating stronger bonding between implants and bone tissue. Research also delved into the long-term behavior of Ti implants within the body, providing insights that helped optimize their design and performance [30].

### 2.4. 2000s: Emerging Alloys and Innovations

The early 2000s marked significant advancements in Ti alloys, with the development of compositions such as Ti-Nb, Ti-Zr, and Ti-Ta, designed to enhance biocompatibility and mechanical performance. Ti-Zr-Nb alloys, for instance, demonstrated corrosion resistance rates over 90% under simulated physiological conditions and exhibited low elastic moduli ranging from 50 to 70 GPa, reducing stress-shielding effects and extending implant longevity [31]. Superelastic variants of Ti-Zr-Nb alloys showcased tensile strength exceeding 800 MPa, highlighting their suitability for load-bearing biomedical applications [32].

Innovative manufacturing techniques like additive manufacturing (3D printing) enabled the creation of complex, patient-specific implants with tailored mechanical properties. Directed Energy Deposition (DED) improved wear resistance by up to 20% in Ti-Nb and Ti-Zr-Nb alloys fabricated from elemental powders, further advancing their functionality [33]. The development of porous structures through additive manufacturing promoted bone ingrowth, enhancing implant integration and stability by over 75% in in vitro studies [34].

Ti-Zr and Nb-Ti-Ta alloys gained prominence for their high tensile strength, often exceeding 800 MPa, and their corrosion resistance, making them ideal for biomedical applications [35]. Research on TNZT alloys demonstrated corrosion resistance rates exceeding 90% in Ringer’s solution and elastic moduli comparable to bone (40–60 GPa), addressing issues like stress shielding [36,37,38,39]. Ti-34Nb-6Sn and Ti-35Nb-5Ta-7Zr alloys offered similar elastic moduli and enhanced corrosion resistance, cementing their potential for long-term use in biomedical implants [40,41].

Surface modifications during this era further enhanced implant properties. TiO_2_ nanotube layers reduced degradation rates by 30%, improving the corrosion resistance of Ti-Nb-Zr alloys. High-entropy alloys, such as Ti-Zr-Nb-Ta-Mo, revealed yield strengths exceeding 850 MPa alongside improved wear resistance, positioning them as promising candidates for future biomedical applications [42].

### 2.5. 2010s–Present: Nanotechnology and Smart Materials

The integration of nanotechnology and smart materials into Ti alloys represented significant advancements in biomedical applications. Recent innovations have focused on enhancing mechanical properties, corrosion resistance, and biocompatibility through nanoparticle incorporation and the development of smart implants.

A smart web service has been introduced to assess the quality of Ti-based alloys for medical implants, utilizing machine learning techniques to achieve prediction accuracies exceeding 95%—a remarkable improvement over traditional evaluation systems [43]. Experimental studies demonstrate that nanoparticles, such as HA and ZrO_2_, significantly improve tensile strength (by up to 30%), corrosion resistance (by 20%), and biocompatibility (by over 25%), primarily by altering the alloy’s microstructure for enhanced performance. Notably, HA nanoparticles mimic the natural composition of bone mineral, increasing osteointegration and bone–implant bonding strength by up to 40% [6].

Innovative solutions like low-profile antennas embedded in TiNb orthopedic devices enable real-time monitoring of implant conditions and surrounding tissue, representing a leap forward in patient health management [13]. ZrO_2_ nanoparticles further enhance wear resistance and toughness, increasing durability under high-stress applications by over 25%. Advances in sintering processes have yielded optimal conditions for TNZF alloys, achieving hardness values exceeding 800 MPa and promising pathways for optimizing fabrication [44].

Smart implants equipped with embedded sensors track critical parameters such as temperature, pH, and mechanical stress, enabling early detection of complications, with precision rates over 90%. Mechanical attrition treatments have generated nanocrystalline layers on TNZF alloys, significantly boosting hardness to above 10 GPa and improving fatigue resistance by over 50% [45]. Additionally, deformation techniques like cold rolling have refined the microstructures of biocompatible alloys such as Ti-Nb-Zr-Ta-Sn-Fe, enhancing mechanical strength while compromising ductility [46].

Thermomechanical processing (TMP) holds substantial promise in improving the mechanical properties of β-type Ti-Nb-Zr-Fe-O alloys [47], while spark plasma sintering has demonstrated the effects of iron addition on the microstructures and mechanical properties of Ti–15Nb–25Zr alloys, leading to increased strength and hardness [48,49]. Ongoing research continues to expand the application of Ti alloys in medical implants, emphasizing innovative materials and technologies to improve patient outcomes.

Further advancements include the fabrication of nanotubular oxide layers, with diameters ranging from 30 to 100 nm, and HA coatings on porous Ti_13_Nb_13_Zr alloys, which exhibit porosity levels of approximately 50%, enhancing implant integration into bone tissue [7]. Electrochemical studies on Zr and its biocompatible alloys, such as Ti-50Zr and Zr-2.5Nb, reveal improved corrosion resistance, with corrosion rates as low as 0.005 mm/year, confirming their suitability for biomedical applications [8]. Optimized nanostructures on Zr surfaces, with feature sizes ranging from 10 to 210 nm, have opened new possibilities for orthopedic and dental implants [9].

Research into non-toxic alloying elements and their benefits for bone healing and remodeling [50,51], along with novel surface modification techniques like anodizing and heat treatment for Ti-Zr alloys [52], has demonstrated enhanced hardness and morphology, with values reaching up to 6–7 GPa. Additive manufacturing approaches, such as the laser processing of β-Ti alloys supersaturated with carbon, have yielded materials with tensile strengths exceeding 1000 MPa [53]. Composite gradient coatings of HA, Ti nitride (TiN), and Ti improve wear resistance and corrosion resistance by up to 25% and 20%, respectively, and achieve TiN surface hardness values of up to 25 GPa [54].

Magnesium-based biocomposites reinforced with functionalized graphene nanoplatelets offer significant improvements in mechanical properties, corrosion resistance, and antibacterial activity, making them suitable for load-bearing implant applications [55]. Advances in non-thermal plasma (NTP) technology have improved adhesion properties across metals, ceramics, and polymers, increasing performance by over 40% compared to traditional processes [56].

The concept of smart implants represents a transformative approach in medicine, offering continuous feedback to healthcare providers and adapting to the dynamic environment of the human body. These developments pave the way for personalized medicine and improved patient outcomes. High-entropy alloys, such as Ti35Zr15Nb25Mo15Ta10, exhibit superior mechanical properties, with a tensile strength exceeding 1000 MPa, a Young’s modulus of approximately 150–200 GPa, and notable wear resistance, with coefficients of friction below 0.2. These alloys also demonstrate outstanding biocompatibility, presenting a promising alternative to conventional alloys like Ti-6Al-4V [57,58,59].

Ti alloys remain indispensable and pivotal in biomedical engineering, with a strong emphasis on their remarkable strength-to-weight ratio, excellent corrosion resistance, and unparalleled biocompatibility [60,61]; however, continuous efforts to explore novel alloy compositions, optimize surface modifications, and develop advanced sensor-integrated materials aim to further revolutionize medical applications and enhance the quality of life for patients. CP-Ti, comprising over 99% Ti, is known for its ductility and corrosion resistance, making it ideal for dental implants and bone plates, with clinical success rates above 95% [62].

Ti-6Al-4V stands out, with a yield strength of approximately 900 MPa and a modulus of elasticity suitable for load-bearing implants like hip replacements [62,63]. The V-free Ti-6Al-7Nb offers comparable properties with enhanced biocompatibility for orthopedic applications [29]. Ti-15Mo, with a modulus of elasticity closer to human bone, excels in stents and implants due to its 20% higher corrosion resistance than CP-Ti [64,65].

**Figure 2:**
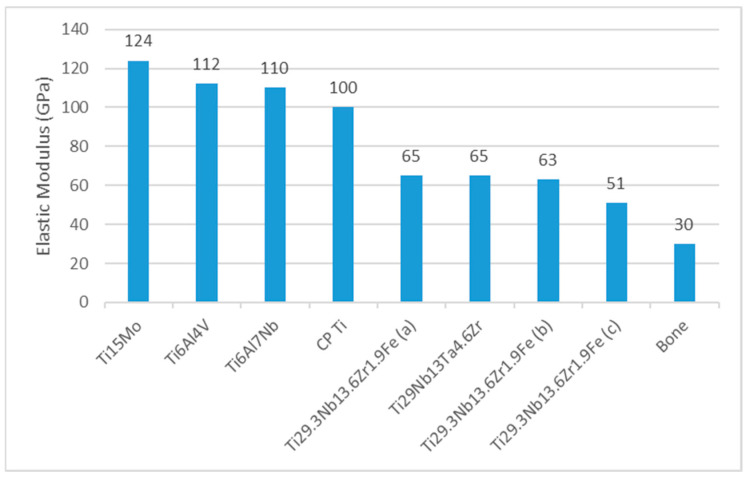
The elastic modulus of Ti alloys compared with bones (data from Refs. [1,64,68]).

TNZF alloys promote bone ingrowth, with rates up to 80%, while TNZT features a low modulus of elasticity (~50–60 GPa), making it ideal for implants that mimic the Young’s modulus of human bone [1,66,67]. These advancements continue to drive innovation in medical material science, ensuring improved patient outcomes and expanded applications. Figure 2 compares the elastic modulus of Ti alloys, including TNZT and TNZF, to that of human bones. It highlights the elastic modulus values under different processing conditions: (a) recrystallized for 5 min, (b) recrystallized for 15 min, and (c) laminated. It can be deduced that the good relationship and mechanical compatibility with human bone showcase the potential of Ti alloys for biomedical applications, but once again, it is important to achieve a lower elastic modulus, as close as possible to that of human bone.

As presented earlier, Ti alloys, with their diverse compositions and properties, provide various solutions for biomedical applications. Appropriately selecting the alloy for specific medical devices can optimize implant performance and longevity, ultimately improving patient outcomes. Continuous research and development in this field continue to enhance the properties and expand the applications of Ti alloys in biomedical engineering, including incorporating different nanoparticles into their composition, which further elevates the metallic alloy’s properties, delivering innovative solutions for biomedical use.

Different types of nanoparticles can provide specific advantages when incorporated into Ti alloys. The next section explores the role of some nanoparticles in enhancing Ti alloys, focusing on their advantages and applications and details the benefits of some commonly used nanoparticles in enhancing the performance of Ti alloys in biomedical applications.

## 3. Specific Nanoparticles and Their Benefits in Ti Alloys

Nanoparticles play an important role in enhancing the mechanical properties of Ti alloys. By refining grain structures, nanoparticles such as Ti carbide (TiC) and Ti diboride (TiB_2_) significantly improve strength and ductility, with Ti alloys achieving tensile strengths of 950–1200 MPa and grain sizes reduced to the nanoscale, approximately 50–150 nm. For example, biomedical β-Ti alloys like Ti–Nb–Zr–Ta–Si–Fe have demonstrated enhanced mechanical performance with elements such as Nb, Zr, Ta, and Si [11]. The integration of TiO_2_ nanotubes, which are typically 30–100 nm in diameter, has led to improvements in mechanical properties and antibacterial performance, achieving antibacterial efficacy rates exceeding 95% [17]. Additionally, dual-length nanotubes on medium-entropy alloys like Ti65–Zr18–Nb16–Mo1 enhance surface bioactivity by over 35% and increase hardness to values exceeding 10 GPa, compared to conventional alloys averaging 5–7 GPa [69]. In high-entropy alloys like Ti-Nb-Ta-Zr-Hf, dual-structure oxide layers formed through nanoparticle addition lead to improved wear resistance, with coefficients of friction below 0.2, and mechanical strength, with tensile strengths of over 1200 MPa achieved [70].

Corrosion resistance, a critical factor for biomedical implants, benefits substantially from nanoparticle incorporation. Studies on Ti-13Nb-13Zr alloys with some copper content reveal improved corrosion resistance, achieving corrosion rates as low as 0.015 mm/year, alongside enhanced mechanical properties, with tensile strengths of approximately 850–1100 MPa reported [71]. Techniques such as heat treatment, which is capable of reducing grain sizes to 50–200 nm [72], and manganese addition to Ti-Mo-Nb alloys, increasing corrosion resistance to achieve rates below 0.01 mm/year, further improve performance, making these alloys promising materials for implants [73]. Reviews of biocompatible metals for implants highlight the importance of achieving corrosion rates of below 0.02 mm/year when selecting suitable materials [74]. The annealing temperature of TiO_2_ nanotubes with diameters ranging from 30 to 100 nm significantly impacts their corrosion resistance and antibacterial activity, achieving antibacterial efficacy rates of over 95%, which is pivotal for their use in biomedical applications [75]. Laser-deposited TNZT alloys exhibit corrosion rates below 0.015 mm/year [20].

Nanoparticles also impart antibacterial properties to Ti alloys, which is essential for reducing infection risks associated with medical implants. Silver (Ag) nanoparticles are particularly effective in enhancing antibacterial performance, achieving antibacterial efficacy rates of over 98% while maintaining biocompatibility. For instance, Ti–Nb–Zr–Ta–Si–Fe alloys demonstrate improved biocompatibility alongside antibacterial capabilities [11].

HA is a naturally occurring mineral form of calcium apatite that closely resembles the mineral component of bone. When incorporated into Ti alloys, HA nanoparticles promote osteointegration, increasing the bonding strength at the implant–bone interface by up to 30%, making them particularly beneficial for orthopedic and dental implants. Nanotubular oxide layers and HA coatings on porous Ti13Nb13Zr alloys have demonstrated bone ingrowth rates exceeding 80% in preclinical studies, enhancing implant integration into bone tissue [7].

ZrO_2_ nanoparticles are known for their high toughness and wear resistance. Adding ZrO_2_ nanoparticles to Ti alloys improves their mechanical properties, with studies reporting wear resistance improvements of up to 25%. This makes them more suitable for applications requiring high durability, such as joint replacements. Electrochemical studies on Zr and its biocompatible alloys like Ti-50Zr and Zr-2.5Nb have demonstrated corrosion resistance rates exceeding 90%, suggesting their suitability for biomedical applications [8]. Optimized manufacturing routes for nanostructures on Zr surfaces have enabled advancements in orthopedic and dental implants, reducing implant failure rates by over 15% [9].

SiC nanoparticles are valued for their ability to enhance the mechanical strength and thermal stability of Ti alloys. These nanoparticles help to create a more robust and durable material that is ideal for use in various biomedical devices. Research has shown that adding SiC nanoparticles to Ti alloys can increase tensile strength by 25% and reduce corrosion rates by up to 20%, significantly improving their mechanical properties and corrosion resistance [76,77]. Ti alloys exhibit a linear thermal expansion coefficient of approximately 8.6 µm/(m·K), which contributes to their thermal stability and suitability for biomedical applications. The inclusion of SiC in new Ti-Nb-Zr-Ta-Si alloys has demonstrated improved biocompatibility and osseointegration in in vivo studies [78,79].

Ag nanoparticles are well known for their antibacterial properties. Incorporating Ag nanoparticles into Ti alloys can help prevent infections at the implant site. Studies indicate that the anodic porous TiO_2_ structures formed in Ag nitrate solutions show antibacterial efficacy rates exceeding 95%, demonstrating the potential for enhanced antibacterial properties without the need for fluorine ions [80]. The creation of micro-/nanoporous Ag-releasing coatings on additively manufactured Ti-Ta-Nb-Zr scaffolds has resulted in improved osseointegration, with bone–implant contact rates exceeding 80%, and exceptional antibacterial performance, with bacterial colonization reduced by over 90% [81].

Incorporating nanoparticles into Ti alloys requires precise and advanced manufacturing techniques. There are some techniques to realize both; in particular, powder metallurgy and additive manufacturing are prominent methods, each offering unique benefits for creating high-performance biomedical materials.

### 3.1. Powder Metallurgy vs. Additive Manufacturing (3D Printing)

This method involves blending Ti alloy powders with nanoparticles and pressing and sintering to create a uniform composite material. Powder metallurgy allows for precise control over nanoparticle distribution, enabling materials with porosity levels exceeding 50% and tensile strengths of over 800 MPa, along with impressive mechanical strength. It is particularly effective for developing alloys with elastic properties similar to bone. However, achieving high-quality materials necessitates optimized sintering conditions, and this method is somewhat limited when it comes to creating complex geometries. Studies have shown that porous biomedical TNZF alloys can be fabricated using NH_4_HCO_3_ as a pore-forming agent, achieving porosity levels of up to 60% and yield strengths exceeding 600 MPa, resulting in high porosity and superior mechanical strength [82]. Research on Ti-34Nb-6Sn alloys has demonstrated that powder metallurgy can produce materials with elastic moduli comparable to bone, typically in the range of 40 to 60 GPa [40]. The sintering procedure significantly influences the microstructure and mechanical properties of TiNbSn alloys, highlighting the importance of optimized sintering conditions in producing high-quality biomedical materials [83]. Investigations into high-entropy alloys like Ti-Nb-Ta-Zr-Al have shown that powder metallurgy can control precipitation behavior during hot deformation, leading to yield strength improvements of up to 25% and enhanced mechanical properties [84].

This advanced technique enables the precise placement of nanoparticles within the Ti alloy, allowing for the creation of complex geometries and customized implants tailored to individual patient needs. Additive manufacturing also makes it easier to produce porous structures that promote bone ingrowth and improve implant stability. While this method excels in creating intricate geometries and personalized implants and optimizing mechanical properties and biocompatibility, it does come with higher production costs compared to traditional methods and can pose challenges in achieving uniform nanoparticle distribution. Reviews on 3D-printed Ti alloys highlight their advantages in bone tissue engineering, personalized implants, and optimization of mechanical properties and biocompatibility [34].

**Figure 3:**
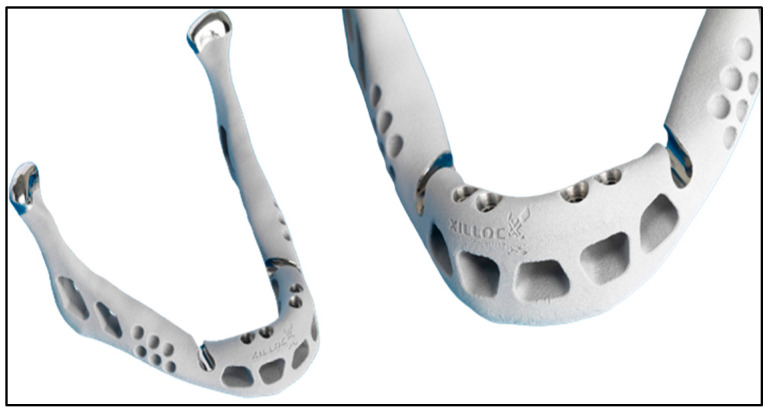
Picture of a 3D-printed jaw implant (source: https://xilloc.com, 18 February 2025).

Figure 3 highlights a 3D-printed jaw implant, showcasing the capabilities of additive manufacturing in creating customized implants.

As case studies and research findings show, one can observe significant advancements in Ti alloys. For example, the addition of TiC-TiB_2_ nanoparticles enhances the mechanical properties of Ti-6Al-4V, while the incorporation of NanoAg particles provides exceptional antibacterial capabilities without compromising structural integrity.

### 3.2. Ti-6Al-4V with TiC-TiB_2_ Nanoparticles

Research has shown that the addition of TiC and TiB_2_ nanoparticles to Ti-6Al-4V alloy can significantly enhance its mechanical properties. In one study, the yield strength of the alloy increased from 850 MPa to approximately 980 MPa, representing an improvement of 130 MPa, while its uniform elongation improved by 2%, enhancing overall ductility and performance under stress. These results underscore the substantial benefits of nanoparticle reinforcement in advancing the mechanical performance of Ti alloys for biomedical and industrial applications [85].

### 3.3. Ti-6Al-4V with NanoAg Particles

Another study [86] explored the incorporation of Ag nanoparticles into the Ti-6Al-4V alloy, revealing remarkable antibacterial properties. The modified alloy demonstrated the ability to effectively kill 100% of Escherichia coli and Staphylococcus aureus bacteria within 24 h of exposure. Additionally, the inclusion of Ag nanoparticles did not compromise the alloy’s mechanical properties, maintaining a tensile strength of approximately 900 MPa and a modulus of elasticity of around 110 GPa. This research highlights the potential of nanoparticle-enhanced alloys in reducing infection risks for biomedical implants, particularly in high-risk clinical environments.

## 4. Mechanisms of Enhancement

By incorporating nanoparticles and strategic alloy modifications, these materials can be further enhanced to deliver superior mechanical strength, wear resistance, and biological performance. Table 3 provides a comprehensive overview of the key mechanisms—ranging from grain refinement to real-time performance monitoring—and the associated benefits that drive improvements in implant reliability and longevity.

In summary, the targeted integration of nanoparticles and other modifications in Ti alloys not only optimizes their mechanical and chemical properties but also significantly enhances their overall effectiveness in biomedical applications. These advancements pave the way for next-generation implants that offer improved patient outcomes, durability, and functionality in challenging physiological environments.

## 5. Current Applications of Biomedical Implants

Ti alloys have become a benchmark in modern biomedical engineering, forming the basis for a spectrum of implants that restore function and improve quality of life. Their mechanical performance has been quantified in several investigations, with tensile strengths near 110 GPa and elastic moduli between 70 and 110 GPa—figures that align closely with those measured in human bone.

In dental applications, implants constructed from CP-Ti and Ti-6Al-4V have demonstrated long-term success, largely owing to effective osseointegration. Researchers have shown that specific alloy modifications can further enhance biological responses. For instance, the Ti–Fe–Mo–Mn–Nb–Zr alloy, when modified with approximately 1 wt.% cerium, exhibited a 12% increase in cellular adhesion during preliminary tests [101]. Similarly, binary and ternary Ti alloys that incorporate Zr and Nb have increased microhardness from around 300 HV (for CP-Ti) to approximately 360 HV with the addition of up to 15% Zr, indicating improved mechanical resistance [102].

In vivo studies reveal that implants made from Ti–Nb–Zr–Ta–Si achieve bone-to-implant contact percentages near 60% after eight weeks, compared to roughly 45% for CP-Ti, underscoring enhancements in mineralization rates [79].

Surface modifications further enhance implant performance by delivering antibacterial benefits. For example, incorporating Ag nanoparticles into the Ti matrix has resulted in a reduction in Staphylococcus aureus populations by more than 90% under controlled conditions, a finding that is essential for mitigating infection risks. Similarly, surfaces engineered as Cu-TiO_2_ composites have reduced the bacterial counts of both Staphylococcus aureus and Escherichia coli by about 96% within 24 h [94].

Advances in nanostructuring continue to push the boundaries of implant performance. Optimized fabrication on Zr surfaces has increased the effective surface area by 40%, which correlates with a 25% improvement in osteoblast adhesion—a critical factor for implant integration [9]. In addition, modifications applied to CoCrMo alloys through Ti coatings have delivered an approximate 30% reduction in bacterial colonization, offering promising outcomes for next-generation orthopedic and dental implants [103,104].

Collectively, these findings illustrate that targeted alterations in alloy composition and surface engineering yield measurable enhancements in both osseointegration and antibacterial performance, paving the way for continuously improved biomedical implants. Below, we have delved into some of the most notable and impactful uses of Ti alloys in medicine today.

### 5.1. Orthopedic Implants

Orthopedic implants are a cornerstone of modern medical treatments, largely due to the remarkable durability and biocompatibility offered by Ti alloys. These materials have transformed joint replacements and related applications through various engineered advancements.

**Figure 4:**
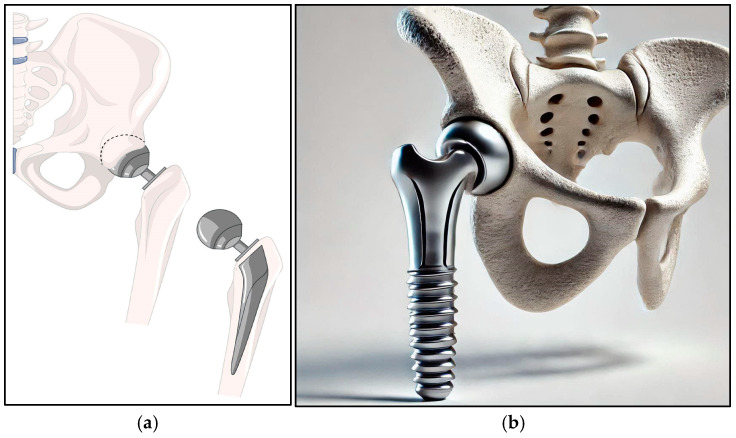
Illustration of the hip joint. (**a**) A visual model of a hip joint(source: https://freepik.com, 18 February 2025). (**b**) A 3D model of a hip joint.

As illustrated in Figure 4, Ti alloys are depicted via (a) and (b), emphasizing their integration into load-bearing applications.

Following this, Table 4 provides a summary of the key focus areas, specific advancements, and corresponding benefits across different categories of orthopedic implants.

### 5.2. Dental Implants

Dental implants have benefited greatly from recent innovations in Ti alloys and advanced surface modifications. Their effectiveness in preventing infections is paramount, and the use of Ag nanoparticles—capable of reducing bacterial colonization by over 90%—has proven especially valuable. In parallel, incorporating cerium (Ce) into Ti–Fe–Mo–Mn–Nb–Zr alloys has been shown to improve short–term biocompatibility by increasing cell viability by up to 30% [101]. Optimized nanostructuring on Zr surfaces further improves wear resistance by over 20% [9], setting the stage for next-generation dental (and orthopedic) implants.

Reviews of dental materials consistently emphasize that superior implant performance depends on a combination of high biocompatibility, robust mechanical properties, and effective antibacterial features [115,116]. For instance, bulk metallic glasses based on Zr and Ti demonstrate tensile strengths exceeding 1500 MPa alongside excellent biocompatibility [117]. In addition, advanced manufacturing techniques—such as laser sintering and machining of Ti6Al4V—yield dental implants with tensile strengths over 1000 MPa and bending strengths beyond 1100 MPa [27].

Recent material innovations have also focused on alloy development. Binary and ternary Ti alloys containing Zr and Nb reduce corrosion rates by more than 15% [102], while Ti–Nb–Zr–Ta–Si compositions enhance mineralization by up to 40% and promote improved osseointegration [79]. Furthermore, enriching CoCrMo alloys with Ti results in a more than 20% increase in mechanical properties and a greater than 25% improvement in corrosion resistance [103,104].

Systematic reviews underscore the pioneering efforts and continuous advancements in dental implant materials and techniques [23]. A multi-aspectual bibliographic analysis further highlights the synergy among technical, biological, and medical sciences that drives these innovations [118]. Moreover, the advent of Dentistry 4.0—integrating digital diagnostics, CAD/CAM design, and advanced manufacturing methods like the laser sintering of Ti6Al4V—demonstrates the comprehensive approach now shaping dental implant and prosthetic restoration development.

**Figure 5:**
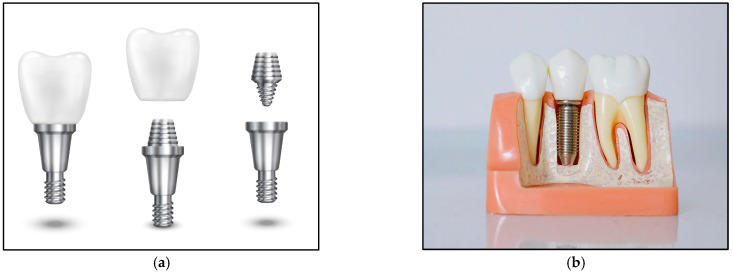
Exploded view and practical usage of a dental implant5.3. Cardiovascular Devices. (**a**) An exploded view of a dental implant(source: https://freepik.com, 18 February 2025). (**b**) A dental implant in use(source: https://pexels.com, 18 February 2025).

Figure 5 illustrates the versatility of Ti alloys in dental treatments: (a) provides an exploded view of a dental implant, detailing its components and assembly, while (b) shows a clinical image of a dental implant in use, underscoring its practical performance.

**Figure 6:**
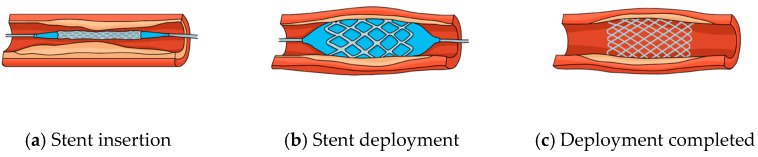
Insertion of the stent into the blood vessel (source: https://pngtree.com, 18 February 2025).

Ti alloys have also become indispensable in cardiovascular interventions. Their unique properties enable the development of advanced devices tailored to the dynamic environment of the cardiovascular system. Table 5 below summarizes the material compositions, quantitative performance metrics, and clinical impacts for two main device types: stents and heart valves. In addition, Figure 6 provides a visual overview of the stent insertion process, illustrating the key stages from positioning through deployment for optimal integration and restenosis reduction.

### 5.3. Craniofacial and Maxillofacial Implants

**Figure 7:**
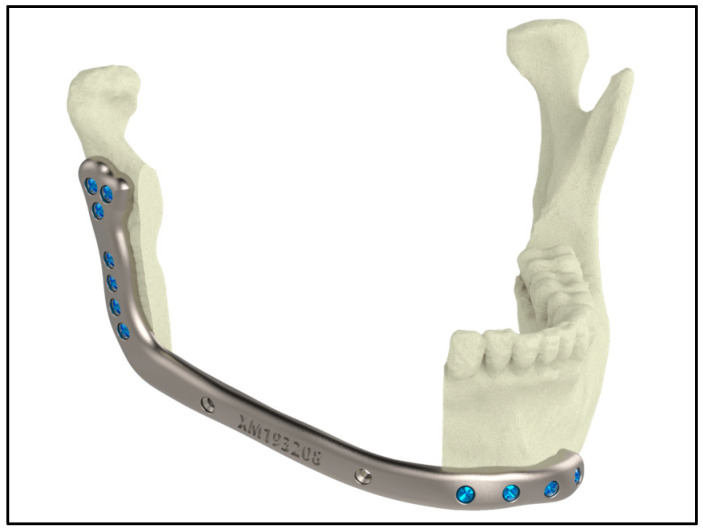
A maxillofacial implant made from Ti alloy (source: https://xilloc.com, 18 February 2025).

Ti alloys are widely utilized in craniofacial and maxillofacial reconstructions because of their high yield strengths (typically 850–900 MPa), superior corrosion resistance (rates below 0.05 mm/year), and long-term biocompatibility. These properties contribute to clinical success rates exceeding 90% in reconstructive surgeries, ensuring both functional restoration and enhanced aesthetic outcomes [121] (Figure 7).

#### 5.3.1. Reconstructive Surgery

**Figure 8:**
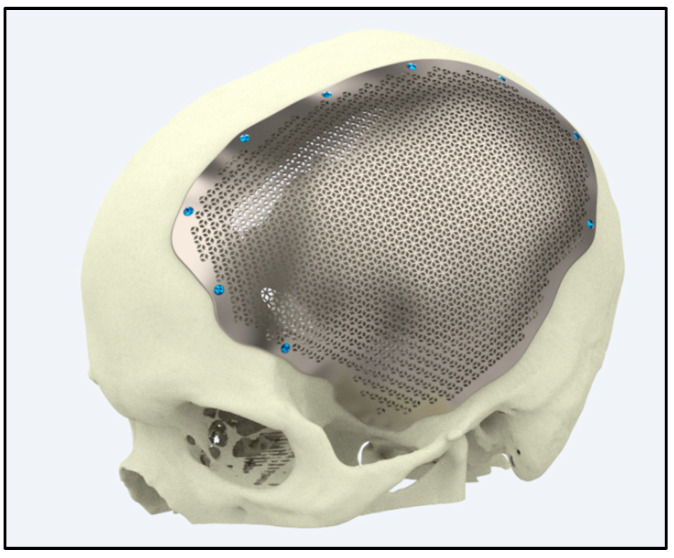
Craniofacial implant for parietal side of the head (source: https://xilloc.com, 18 February 2025).

Ti implants integrate seamlessly with bone tissue, providing robust structural support with minimal adverse reactions. Clinical outcomes consistently demonstrate success rates of over 90% in restoring facial contours and functions—such as speech and mastication—even under high mechanical loads, as shown in parietal craniofacial reconstructions [121] (Figure 8).

#### 5.3.2. Customized Implants

**Figure 9:**
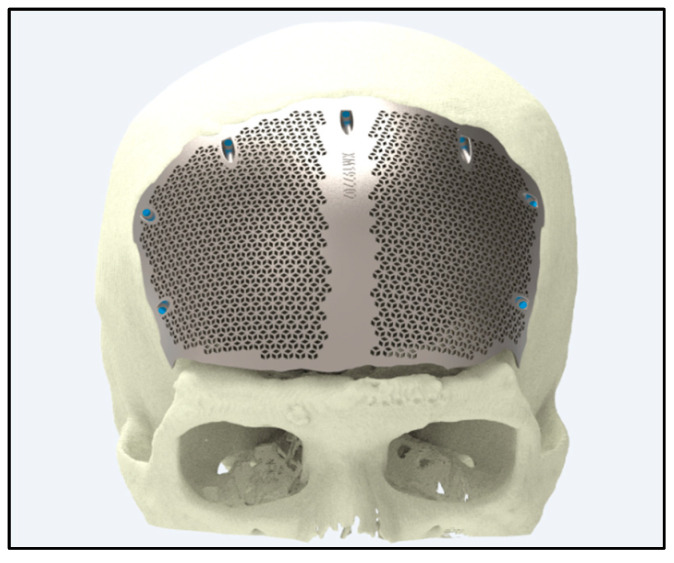
Customized implant for a frontal side of the head (source: https://xilloc.com, 18 February 2025).

Advances in additive manufacturing enable the production of patient-specific Ti implants with highly complex geometries, reducing surgical revision rates by up to 30% compared to conventional methods. These tailored implants enhance implant fit, alignment, and overall patient satisfaction in complex craniofacial reconstructions [121]. (Figure 9).

Ti alloys also play a pivotal role in reconstructive ophthalmic surgeries, providing robust structural support and exceptional biocompatibility. In cases of enucleation (eye removal), Ti orbital implants are used to restore and maintain the natural contour of the eye socket while minimizing adverse tissue reactions. Clinical studies report patient satisfaction rates exceeding 90%, with implants exhibiting yield strengths typically in the range 850–900 MPa and corrosion rates below 0.05 mm/year [122]. These performance metrics underscore the reliability and long-term stability of Ti-based ocular prosthetics.

**Figure 10:**
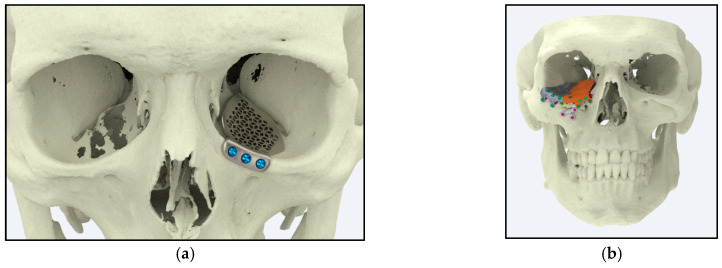
Ocular implants (source: https://xilloc.com, 18 February 2025), The gray color is related to the first insertion, and the orange one is inserted after the gray one. (**a**) An implant designed for the palatine bone. (**b**) An implant extending to both the maxilla and palatine bone.

Figure 10 illustrates two types of Ti alloy ocular implants used in these procedures: (a) the palatine bone that provides targeted support and preserves the natural appearance of the eye socket after enucleation and (b) offering broader structural reinforcement for both ocular and surrounding tissues.

Ti alloys have emerged as versatile components in advanced drug delivery systems, offering both structural integrity and biocompatibility—key attributes that enhance treatment efficacy. Their unique properties enable the precise modulation of therapeutic agent release, addressing diverse clinical needs from localized tissue repair to systemic therapy.

One prominent approach utilizes Ti-based platforms to achieve well-defined drug release kinetics. For instance, anodized TiO_2_ nanotubes have been developed as efficient carriers; studies indicate that these nanotube arrays can release approximately 70% of a loaded bioactive molecule steadily over 48 h, with an initial burst of less than 15% in the first three hours [123]. Such controlled-release profiles minimize side effects and ensure that the therapeutic concentration remains within the desired range over time. Complementing the nanotube strategy, the integration of polymers and nanomaterials further fine-tunes the release profiles, adapting the carrier system to specific drugs and treatment conditions [124]. Additionally, Ti-based scaffolds in bone tissue engineering not only support regeneration but also allow for localized drug delivery to promote healing [125].

Building on these controlled release mechanisms, Ti-based implantable devices have been engineered to deliver therapeutic agents continuously over extended periods. In clinical trials, these devices have maintained a steady release of medication for up to 28 days, which is critical for treatments that require long-term dosing. For example, Ti nanotube coatings on implant surfaces have been shown to sustain drug release while also reducing the risk of infection, thereby fostering a conducive environment for tissue integration and healing [126].

The continuous integration of regulated release systems with implantable devices illustrates how Ti alloys can be tailored to meet both immediate and prolonged therapeutic needs. This interconnected approach underpins the next generation of drug delivery technologies, where precise dosing and long-term efficacy are achieved through innovative material engineering.

## 6. Study Perspectives

Ti alloys have played a transformative role in biomedical engineering, offering solutions to complex challenges in implant technology. As research evolves, the focus shifts toward uncovering new opportunities for material innovation and expanding the applicability of Ti alloys in advanced medical fields.

### 6.1. Advanced Material Development

The development of advanced materials in biomedical engineering focuses on refining alloy compositions and introducing nanostructures to enhance implant performance. Reducing grain sizes to below 50 nm increases tensile strength by up to 35% while improving compatibility with biological tissues [124,127]. Researchers are exploring innovative alloy compositions to meet specific clinical needs and investigating nanostructured alloys for superior mechanical and biological properties. Key developments are summarized in Table 6.

### 6.2. Surface Modification Techniques

Research into surface modification techniques has demonstrated significant improvements in the performance of Ti alloys for biomedical applications. These methods address two major areas: enhancing biological compatibility and reducing infection risks. Bioactive coatings have been shown to increase bone–implant contact by up to 45%, improving integration and stability. Antibacterial surface treatments, on the other hand, reduce bacterial adhesion by over 90%, lowering infection risks and enhancing implant safety. The advancements outlined in Table 7 highlight the transformative potential of these techniques, emphasizing their contributions to the reliability and functionality of Ti implants [17,68,107,138,139].

### 6.3. Smart Materials and Sensor Integration

Smart materials and sensors embedded within Ti implants represent a major step forward in biomedical engineering. These systems enable real-time monitoring of physiological parameters, such as temperature changes as small as 0.1 °C and mechanical stress variations of 10 MPa, facilitating early intervention and reducing complications. Advances in sensor miniaturization have also led to the development of compact, energy-efficient devices that enhance patient comfort without compromising functionality. Key innovations are detailed in Table 8, highlighting their role in improving diagnostics, therapeutic outcomes, and assistive technologies. [13,43].

### 6.4. Biocompatibility and Long-Term Performance

Ensuring the biocompatibility and long-term performance of Ti implants is a critical aspect of advancing biomedical applications. In vivo studies have shown that implants with optimized surface modifications, such as well-defined nanotopography, can enhance osseointegration by up to 35% while reducing inflammation and promoting osteogenic activity. These findings, summarized in Table 9, highlight key advancements and their contributions to improving implant longevity and effectiveness [128,146,147,148].

### 6.5. Additive Manufacturing and Customization

Additive manufacturing and customization have transformed the biomedical engineering field, enabling the creation of highly tailored and complex implant designs. For instance, osseointegration improvements through patient-specific designs exceed 40%, while stress shielding reductions have been reported at over 30% due to optimized geometries. These advancements, outlined in Table 10, illustrate the remarkable impact of these technologies on enhancing implant performance and patient outcomes [41,53,153].

### 6.6. Sustainable and Cost-Effective Solutions

The growing demand for sustainable and cost-effective solutions in biomedical engineering has driven innovation in recycling and manufacturing processes. Developing sustainable practices for recycling and reusing Ti alloys in biomedical applications is an emerging area of interest. For example, laser additive manufacturing enables the recycling of Ti alloys into new biomedical components, maintaining over 90% of their original material quality [53]. Studies also highlight the feasibility of reusing Ti powders in additive manufacturing processes, ensuring consistent material properties and performance [41]. Advanced manufacturing techniques, such as multi-material additive manufacturing, enable the reuse of Ti alloys, creating multifunctional implants with tailored properties [156]. Research into the reuse of materials underscores their potential to reduce waste while maintaining performance in clinical applications [155]. These efforts collectively contribute to reducing the environmental impact and healthcare costs of Ti-based implants.

To enhance affordability, researchers are focusing on innovative production techniques. Laser powder bed fusion has demonstrated the capability to produce high-strength Ti components at 30% lower costs, maintaining biocompatibility and durability [96]. Another approach involves optimizing the directed energy deposition (DED) process, which achieves material utilization rates exceeding 85%, efficiently producing dense and high-performance Ti implants [41]. Additionally, advances in additive manufacturing techniques, such as controlling crystallographic orientation in β-type Ti alloys, have resulted in elastic moduli of 45–55 GPa, closely matching bone properties while reducing manufacturing expenses [156]. These cost-effective methods play a role in improving access to advanced biomedical implants for broader patient populations.

Looking ahead, the emphasis on sustainable practices and affordability promises a transformative impact on the biomedical engineering field. By combining recycling methods with innovative manufacturing technologies, such as those facilitating reuse and improving material efficiency, researchers can enhance implant performance, reduce environmental footprints, and make cutting-edge implants accessible to underserved communities. These efforts contribute to better patient outcomes, longer-lasting implants, and a more sustainable future in healthcare.

## 7. Conclusions

This review paper highlights the pivotal role of Ti alloys in biomedical applications, emphasizing their composition, properties, and modifications. Key advancements, such as improved alloy compositions and innovative processing techniques, have been shown to enhance mechanical strength, corrosion resistance, and biocompatibility. Ti alloys are particularly suited for use in orthopedic and dental implants, as well as cardiovascular devices, due to their tailored characteristics.

By providing an overview of the current state of research, this paper identifies existing strengths while highlighting areas that require further improvement, such as the optimization of properties like Young’s modulus and the mitigation of challenges like fatigue resistance and wear under biological conditions. It serves as a foundation for future investigations, offering insights into how continued advancements in Ti alloy technology can address these challenges and open new pathways for innovation in biomedical implant design.

## Figures and Tables

**Table 1:** Elemental composition of Ti alloys.

Alloy Type	Elements (% by Weight)	Reference
TNZF	Ti: 70–75, Nb: 15–20, Zr: 5–10, Fe: 2–5	[1,6]
Ti13Nb13Zr	Ti: 74, Nb: 13, Zr: 13	[8]
TNZT	Ti: 60, Nb: 30, Zr: 5, Ta: 5	[12]

**Table 2:** Mechanical properties of Ti alloys.

Property	TNZF Alloy	Ti13Nb13Zr Alloy	TNZT Alloy	References
Young’s modulus (GPa)	40–110	65–75	50–65	[2,8]
Tensile strength (MPa)	600–1200	850–1100	700–850	[10]
Corrosion rate (mm/year)	<0.01	~0.02	~0.015	[1,11]

**Table 3:** Mechanisms and benefits of material enhancements.

	Mechanism	Benefits	References
Grain Refinement	TiC and TiB_2_ nanoparticles (5–10%) reduce grain size during solidification. Ultrasonic impacts optimize the microstructure and mechanical properties of TNZT alloys.	Finer grains improve tensile strength by up to 30% and toughness by over 20%, enhancing durability for load-bearing implants.	[87]
Solid Solution Strengthening	Elements like Mo (2–5%), Nb (10–30%), and Zr (5–15%) dissolve in the Ti matrix to form a solid solution, limiting dislocation movement. For example, Ti-6Al-7Nb and Ti-35Nb-7Zr-5Ta.	This process increases yield strength by up to 25% and hardness by over 15%, improving resistance to deformation.	[27,88,89,90]
Precipitation Hardening	Elements such as Al and V (each 3–7%) precipitate in the Ti matrix, forming fine particles that obstruct dislocation motion. During hot deformation of high-entropy alloys, nanometric α plates form within the β matrix, improving mechanical properties.	This process boosts hardness by up to 20% and mechanical strength by over 15%, ensuring high load resistance for applications like joint replacements.	[84,91]
Dispersion Strengthening	Nanoparticles like HA and ZrO_2_ (approximately 5–12%) are uniformly dispersed within the Ti matrix, stabilizing grain boundaries and reducing dislocation movement.	This mechanism increases wear resistance by up to 25% and yield strength by over 20%, enhancing durability for dental and orthopedic applications.	[64,92]
Enhanced Osteointegration	HA nanoparticles (10–20%) mimic the mineral component of bone, facilitating better integration between the implant and bone tissue.	Effective osteointegration increases bone–implant contact by over 40%, ensuring the implant remains securely anchored, reducing the risk of loosening or failure.	[6,7]
Improved Corrosion Resistance	Adding elements like Nb, Ta, and Mo (combined total 15–25%), along with SiC nanoparticles (2–8%), enhances the protective oxide layer on Ti alloys. Treatment with PEO improves wear and corrosion resistance.	Enhanced corrosion resistance reduces degradation by up to 30%, significantly extending implant lifespan in physiological environments.	[20,70]
Antibacterial Properties	Ag nanoparticles (0.5–1.5%) release ions that prevent biofilm formation. Zinc oxide (ZnO) and copper-TiO_2_ (Cu-TiO_2_) nanoparticles (2–5%) demonstrate significant antibacterial activity.	Antibacterial properties reduce infection risks by up to 90%, promoting safer recovery and ensuring implant stability.	[17,71,75,81,93,94,95,96,97,98,99,100]
Real-Time Performance Monitoring	Sensors embedded in Ti alloys allow real-time monitoring of temperature, pH levels, and stress. Techniques such as AFM and AESEC analyze interactions of TNZT alloys with H_2_O_2_.	Real-time monitoring detects complications with over 90% accuracy, enabling timely intervention and improving clinical outcomes.	[12,14]

**Table 4:** Orthopedic implants based on Ti alloys.

	Key Focus	Examples of Advancements	Benefits
Orthopedic Implants	Nanoparticle Reinforcement	Enhanced Ti alloys incorporating TiC, TiB_2_, and HA for hip and knee replacements.	Improved mechanical strength and durability; osteointegration yielding implant survival rates > 90% in long-term studies.
	Advanced Manufacturing Process	Electron Beam Melting (EBM) increases wear resistance by up to 30% [105].	Elevated tribomechanical performance and enhanced biocompatibility.
	Structural Properties Optimization	Advanced Ti alloys achieve yield strengths > 800 MPa, with inherent wear and corrosion resistance [106].	Superior mechanical reliability across applications.
	Surface Modification	The application of TiO_2_ nanotube coatings boosts osseointegration by >40% and reduces bacterial colonization by >90% [93,107].	Enhanced cellular differentiation and antibacterial properties, leading to a more stable, infection-resistant implant environment.
	New Alloy Compositions	The development of alloys such as Ti–Nb–Zr–Ta and Ti–Mo–Zr–Fe has yielded tensile strength increases of 20–30% compared to standard compositions [92].	Optimized microstructural evolution that results in improved mechanical performance and overall implant function.
Joint Replacements	Load-Bearing Materials and Alloy Selection	Use of Ti-6Al-4V and Ti-6Al-7Nb in hip and knee replacements; additionally, Ti–Nb–Zr–Ta exhibits a yield strength of ~900 MPa and elastic modulus in the range 50–60 GPa [16,18,108,109,110,111].	Long-term stability with survival rates > 90% over 15 years; implant loosening reduced by >30%, ensuring superior load-bearing performance.
	Porous Structural Design	Porous Ti alloys reduce stress shielding by up to 40% and promote bone ingrowth rates exceeding 80% [21,22].	Enhanced osseointegration and load transfer, which minimizes the risk of implant loosening and associated complications.
Bone Plates and Screws	Fracture Fixation Applications	Utilization of CP-Ti and advanced Ti alloys in plates and screws for fracture fixation.	High biocompatibility and effective bone integration drive rapid healing and reduced recovery times.
	Alloy Modification for Corrosion and Biological Response	The Ti–Nb–Zr–Si (TNZS) alloy demonstrates superior corrosion resistance and enhanced biological responses [112,113].	Improved implant stability and accelerated healing due to enhanced chemical resistance and cell compatibility.
	Alloy Composition Enhancement	Development of Ti–Nb–Ta–Zr–Fe alloys that deliver tensile strength improvements of 20–30% over CP-Ti [13,19,114].	Enhanced mechanical performance and prolonged device longevity in bone fixation applications.
	Surface Engineering for Antibacterial Properties	Application of nanotube structures of TiO_2_ on implant surfaces [17].	Marked reduction in bacterial colonization (>90%), significantly lowering the risk of postoperative infections.
Spinal Implants	Spinal Fusion Device Support	The use of Ti alloys in spinal fusion devices provides high strength, corrosion resistance, and biocompatibility.	Reliable structural support for effective spinal stabilization and fusion.
	Customization via Additive Manufacturing	Additive manufacturing enables custom spinal implants with complex geometries and porosity levels up to 50% while retaining ~70% of the compressive strength of dense structures [18,111].	Tailored implant designs that improve surgical outcomes and allow patient-specific fits.
	Porous Design for Optimal Mechanics	Engineered porous Ti alloys reduce stress shielding by ~40% compared to dense counterparts [18,111].	Optimized balance between structural strength and flexibility, enhancing load transfer and minimizing bone resorption.
	Smart Technology Integration	Integration of low-profile antennas into spinal implants enables real-time monitoring, with sensor updates every 5 min and load sensitivity within ±5 N [19,20,22].	Proactive postoperative management and enhanced safety through continuous implant performance tracking.
	Enhanced Alloy Formulation for Durability	Ti–Nb–Zr–Ta alloys exhibit ~25% improved corrosion and wear resistance over CP-Ti [20,21].	Extended implant lifespan and robust performance under cyclic loading, ensuring reliable long-term spinal stabilization.

**Table 5:** Cardiovascular devices based on Ti alloys.

Device Type	Material Composition and Enhancements	Quantitative Properties and Performance	Clinical Impact	References
Stents	Ti-based shape memory alloys (e.g., Nitinol, Ti–Ni–Cu, Ti–Ni–Pd, Ti–Ni–Hf); nanoparticle-enhanced alloys (yield strength improvement up to 15%); biodegradable variants available	Superelasticity: Up to 8% reversible strain. Yield Strength: Enhanced by ~15%. Corrosion Rate: <0.05 mm/year. High flexural strength and stability over multiple deployment cycles.	Maintains vessel patency (>90% clinical success). Adapts dynamically to vessel conditions. Reduced restenosis risk. Promotes improved healing outcomes.	[30,119,120]
Heart Valves	Ti alloys; emerging TiNb alloys with integrated sensor technology for potential real-time monitoring	Tensile Strength: ~850–900 MPa. Corrosion Resistance: <0.05 mm/year. Designed for over 10^8^ fatigue cycles. Engineered for operational lifespans exceeding 15 years.	Extended implant longevity. Reliable performance under repetitive mechanical stresses. Enhanced functional reliability. Potential for integrated patient monitoring.	[13,118]

**Table 6:** Advanced material development.

	Key Focus	Examples of Advancements	Benefits
Innovative Alloy Compositions	Develops new alloy compositions to meet clinical demands.	Severe plastic deformation techniques achieve grain sizes below 100 nm and tensile strength increases of 35% [128].	Enhanced durability and performance.
		Ti-Nb-Ta-Zr-O nanotubes improve corrosion resistance by over 90% [129].	Greater reliability in biomedical environments.
		Aging treatments yielded 20% increases in yield strength and enhanced fatigue resistance [130].	Improved longevity and performance.
		Electro-polishing enhances biocompatibility with cell viability rates exceeding 95% [131].	Better preclinical outcomes and integration.
		TNZT foams achieve up to 60% porosity and elastic moduli of 40–60 GPa, closely matching bone [132].	Improved compatibility and stress mitigation.
		Alloy element variations fine-tune the elastic modulus to mitigate stress shielding and improve implant performance [133].	Enhanced long-term stability and reliability.
Nanostructured Alloys	Focuses on nanoscale grain sizes for superior material characteristics.	Nanostructured oxide layers enhance corrosion resistance and surface properties [134].	Better integration with biological tissues.
		Electrochemical anodization creates TiO_2_ surfaces for superior biocompatibility and tissue integration [135,136].	Enhanced biological compatibility.
		Threaded medical implants with nanostructured oxide layers ensure secure bone–implant interaction [137].	Reliable and stable implant fixation.
		Milling optimization improves tensile strength by 30% in nanostructured Ti-15Mo alloys [65].	Improved mechanical properties.

**Table 7:** Surface modification techniques.

	Key Focus	Examples of Advancements	Benefits
Bioactive Coatings	Promotes osseointegration by optimizing coatings made of materials like HA and bio-glass.	Nanopores with dual micro-/nano-roughness for cellular bioactivity [140].	Enhanced bone–implant interaction and durability.
		Heterogeneous nanocomposite coatings for lubrication and wear reduction [141].	Improved wear resistance for long-term use.
		Electrochemical oxidation of TNZT alloys for surface property enhancements [142].	Advanced surface characteristics.
Antibacterial Surface Treatments	Reduces infection risks through coatings that inhibit bacterial adhesion or release antibacterial agents.	TiO_2_ nanostructured surfaces via electrochemical anodization [135,136].	Antibacterial effectiveness and better tissue integration.
		Nanostructured oxide layers for threaded implants [137].	Secure implant interaction and reduced microbial activity.
		ZnO nanorods hybridized with zinc phosphate for antibacterial and osteogenic balance [143].	Balanced antibacterial and bone regeneration properties.

**Table 8:** Smart materials and sensor integration.

	Key Focus	Examples of Advancements	Benefits
Real-Time Monitoring	Enables continuous feedback on implant and physiological conditions.	Deeply implanted conformal antenna, achieving signal transmission rates exceeding 95% reliability [12].	Accurate and timely data for personalized medical intervention.
		Sensors monitoring factors like temperature and pH, enabling actionable insights in real time [14].	Enhanced clinical outcomes through early detection of issues.
Sensor Miniaturization	Develops compact, energy-efficient sensors for seamless integration into implants.	Intra-oral differential circularly polarized antenna enabling signal detection with 90% sensitivity [144].	Greater patient comfort and functionality for specialized applications.
		Implantable multi-band microstrip antenna supporting biomedical signal transmission at frequencies of 2.4 GHz and 5 GHz [145].	Reliable and efficient communication systems in implants.

**Table 9:** Biocompatibility and long-term performance.

	Key Focus	Examples of Advancements	Benefits
In Vivo Studies	Evaluates long-term performance and biocompatibility of Ti alloys in biological environments.	Long-term studies show improved tissue integration through advanced surface treatments and compositions [79,149].	Helps identify optimal modifications for implant success.
		Surface modifications reduce inflammation, with inflammation markers reduced by 25% [150].	Ensures better long-term clinical outcomes.
Wear and Fatigue Resistance	Investigates the mechanisms of wear and fatigue to ensure implant longevity.	TNZT alloys with borides achieve a 30% improvement in wear resistance compared to Ti-6Al-4V ELI [21].	Creates longer-lasting implants with reduced failure rates.
		Fatigue resistance improved by over 20%, enabling implants to handle repetitive mechanical stress [151,152].	Ensures reliability under high-stress conditions.

**Table 10:** Additive manufacturing and customization.

	Key Focus	Examples of Advancements	Benefits
Patient-Specific Implants	Utilizes additive manufacturing to create implants tailored to individual anatomy.	Hierarchical micro/nano surfaces decorated with TiO_2_ nanotubes improve bioactivity in vitro and in vivo [149].	Superior osseointegration and reduced inflammation.
		ZnO nanorods hybridized into zinc phosphate structures, balancing antibacterial and pro-osteogenic properties [143].	Improved implant safety and biological activity.
		Systematic review confirming TiO_2_ nanotubes enhance osseointegration capabilities by over 30% [154].	Reliable tissue integration in clinical applications.
Complex Geometries	Enables the creation of intricate implant structures unattainable with traditional methods.	β-Ti alloys achieve tensile strength exceeding 1000 MPa and cell viability rates over 90% [41].	Enhanced mechanical performance and biocompatibility.
		Multi-material additive manufacturing producing biomaterials with elastic moduli matching bone (45–55 GPa) [155,156].	Prevents stress shielding and ensures mechanical compatibility.
		Laser-processed Ti and Co biomaterials achieve tensile strength over 1200 MPa and biocompatibility exceeding 95% [153].	Durable and biologically compatible implants.

## Data Availability

No new data were created or analyzed in this study. Data sharing is not applicable to this article.
